# Self-Heating Effects In Polysilicon Source Gated Transistors

**DOI:** 10.1038/srep14058

**Published:** 2015-09-09

**Authors:** R. A. Sporea, T. Burridge, S. R. P. Silva

**Affiliations:** 1 Advanced Technology Institute, University of Surrey, Guildford, Surrey, GU2 7XH, United Kingdom

## Abstract

Source-gated transistors (SGTs) are thin-film devices which rely on a potential barrier at the source to achieve high gain, tolerance to fabrication variability, and low series voltage drop, relevant to a multitude of energy-efficient, large-area, cost effective applications. The current through the reverse-biased source barrier has a potentially high positive temperature coefficient, which may lead to undesirable thermal runaway effects and even device failure through self-heating. Using numerical simulations we show that, even in highly thermally-confined scenarios and at high current levels, self-heating is insufficient to compromise device integrity. Performance is minimally affected through a modest increase in output conductance, which may limit the maximum attainable gain. Measurements on polysilicon devices confirm the simulated results, with even smaller penalties in performance, largely due to improved heat dissipation through metal contacts. We conclude that SGTs can be reliably used for high gain, power efficient analog and digital circuits without significant performance impact due to self-heating. This further demonstrates the robustness of SGTs.

Thin-film transistor-based large area electronics have a long and successful history^1^,^2^,^3^,^4^. Simple circuits and arrays of display elements have been produced with increasing sophistication, area, density and performance for several decades^5^. Recently, advances in technology have permitted rapid performance gains of research devices^6^,^7^,^8^ and a diversification of applications attempted^9^,^10^,^11^,^12^,^13^,^14^,^15^,^16^,^17^,^18^,^19^,^20^. This is in part due to fabrication refinements, but mainly a result of completely new processes^18^,^21^,^22^,^23^,^24^,^25^,^26^,^27^,^28^,^29^,^30^,^31^,^32^, substrate materials^33^,^34^,^35^ and semiconductors^26^,^36^,^37^,^38^,^39^,^40^,^41^,^42^,^43^,^44^,^45^,^46^.

Throughout this surge in complexity, speed and extent of applicability, the fundamental device, the thin-film field effect transistor (TFT FET), has witnessed several proposed developments^25^,^38^,^40^,^47^,^48^,^49^,^50^,^51^,^52^,^53^,^54^ with a wide range of practicality, yet its operation has remained essentially unchanged. With the introduction of new material systems, challenges such as contact effects have become increasingly more obvious, and efforts have been devoted to understanding and addressing them^55^,^56^,^57^,^58^,^59^,^60^.

One device which uses contact effects constructively is the source-gated transistor (SGT)^61^,^62^,^63^,^64^,^65^. It is a type of field-effect device realized in a very similar manner to conventional TFTs. The main functional difference is a consequence of the deliberate introduction of a potential barrier at the source electrode, usually through the use of a Schottky contact: when the source barrier is reverse-biased by the applied drain voltage in normal operation, the depletion region at the contact extends across the semiconductor forming a pinch-off region at the edge of the source closest to the drain. This results in low saturation voltage and, with proper design^66^, in output characteristics independent of drain voltage in saturation. From an application point of view, this allows very high gain to be achieved starting at low voltages, for energy-efficient operation^67^.

A number of other properties arise from the current-control method (at the source, as opposed to within the semiconducting channel): tolerance to some geometrical variations, stability under bias stress, but also drain current which is unavoidably lower than that of a FET with the same geometry but with an ohmic source contact.

Furthermore, the current in a Schottky barrier SGT is potentially highly dependent on temperature^68^,^69^,^70^,^71^,^72^,^73^. High sensitivity integrated temperature sensors are possible applications^74^,^75^,^76^, but for generic use, where temperature dependence should be low, design decisions can be taken to minimize drain current temperature coefficient (TC)^77^. In both situations, however, TC is positive. In principle, self-heating during normal operation could lead to performance degradation, or even catastrophic failure through thermal runaway.

In this paper we investigate self-heating effects in Schottky-barrier source-gated transistors. Multi-physics numerical simulations on low-barrier, high current SGTs provide insight into device operation and design strategies for optimization. Measurements on polysilicon SGTs confirm our findings.

The functional features of source-gated transistors make them well suited to specific applications at strategic points in circuit design. Their value is augmented by the fact that their technology is very similar to conventional TFT FETs, allowing the fabrication of SGTs and TFT with very small changes to the process. In practice, circuits including both types of devices would make the most of the characteristics of each: the high speed segments would be designed with FETs (high current devices), while the low power, high gain, or high matching requirement sub-circuits would use the SGT (high gain, geometrically tolerant device) as the main component. By understanding and providing solutions to the limitations of the SGT architecture, the overall performance and reliability of future circuits can be improved, at a time when a significant increase in complexity and environmental constraints are projected for upcoming TFT applications.

## Results

**Figure 1:**
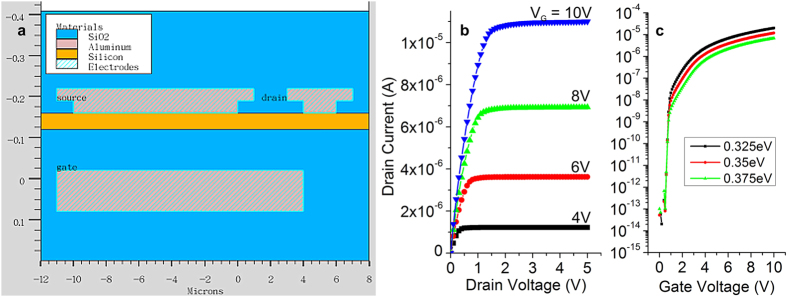
Source-gated transistor (SGT) structure and characteristics. (**a**) Cross section of the SGT defined in Silvaco Atlas for 2D electrical and multi-physics simulation; (**b**) Typical output characteristic of the simulated SGTs showing flat saturation characteristics and low saturation voltage, for source barrier height ϕ_B _= 0.35 eV; (**c**) Transfer characteristics of simulated SGTs for three values of potential barrier at the source metal-semiconductor contact, ϕ_B_, (calculated here as the difference between the conduction band energy and the Fermi level of the metal). For equal steps in barrier height change, the curves are equally spaced on the logarithmic Y scale.

Two dimensional simulations of a conventional bottom-gate, top-contact polysilicon SGT were performed with the Silvaco TCAD suite. The structure’s cross-section is shown in Fig. 1a, with its corresponding output (Fig. 1b) and transfer characteristics (Fig. 1c). Flat saturated output curves, early saturation, and supra-linear dependence of drain current on nominal barrier height are observed.

**Figure 2:**
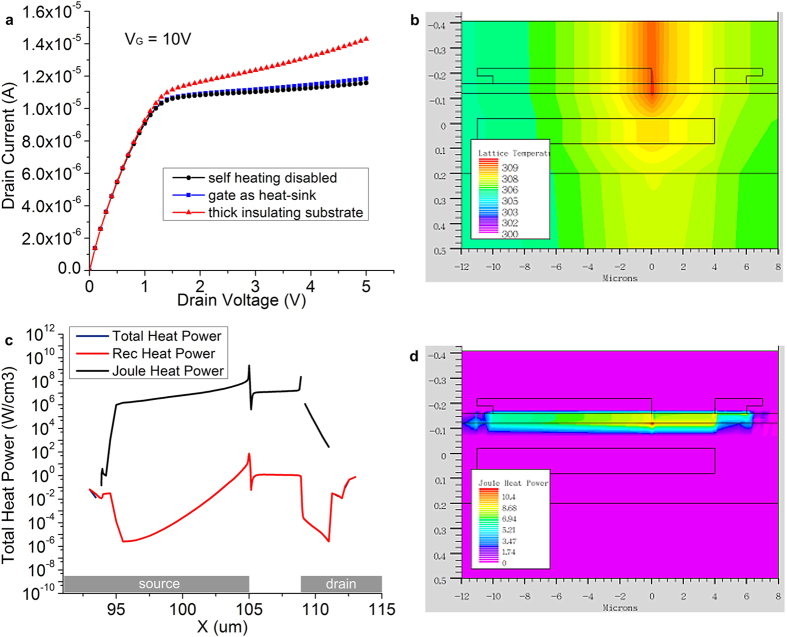
Self-heating in poly-silicon source-gated transistors for (*V*_*G *_= 10 V, *V*_*D *_= 5 V, ϕ_B _= 0.35 eV). (**a**) Output characteristic showing the effect of self-heating with no heat-sink and with the gate acting as heat-sink; (**b**) Cross-section of SGT device and temperature distribution when a 25 μm-thick, low thermal conductivity plastic substrate is used and none of the electrodes are designated as heat sinks (worst case, hypothetical scenario). Peak temperature is localized around the edge of the source contact closest to the drain; (**c**) cut-line of heat generation taken 10 nm into the semiconductor at the insulator interface. The Joule (resistive) power dissipation dominates greatly and losses due to recombination are proportionally small; (**d**) device cross section with power generation overlay (logarithmic scale). All power generation takes place in the semiconductor. The data for other materials results from meshing and is spurious^89^.

The effect of enabling self-heating (SH) in the simulator (lattice heating) is shown in Fig. 2a, in which one output curve was traced for three cases: (I) SH disabled, (II) SH enabled with gate as heat sink, and (III) SH enabled, with only the base of the device (bottom of 10 μm-thick polymer film) as heat sink. Figure 2b shows the temperature distribution for case III, which is the worst-case scenario. For this biasing condition (high VG and high VD), the hot spot is approximately 9 K higher than ambient temperature and is located at the edge of the source closest to the drain. A horizontal cross-section of heat generation at the semiconductor-insulator interface is given in Fig. 2c. For practical purposes, heat power consists entirely of Joule (resistive) heating. The full map of heat generation in the semiconductor (Fig. 2d) shows that it is concentrated around the depletion region at the source edge, with some contribution from the accumulated channel region at the insulator interface and at the extremities of the source and drain.

**Figure 3:**
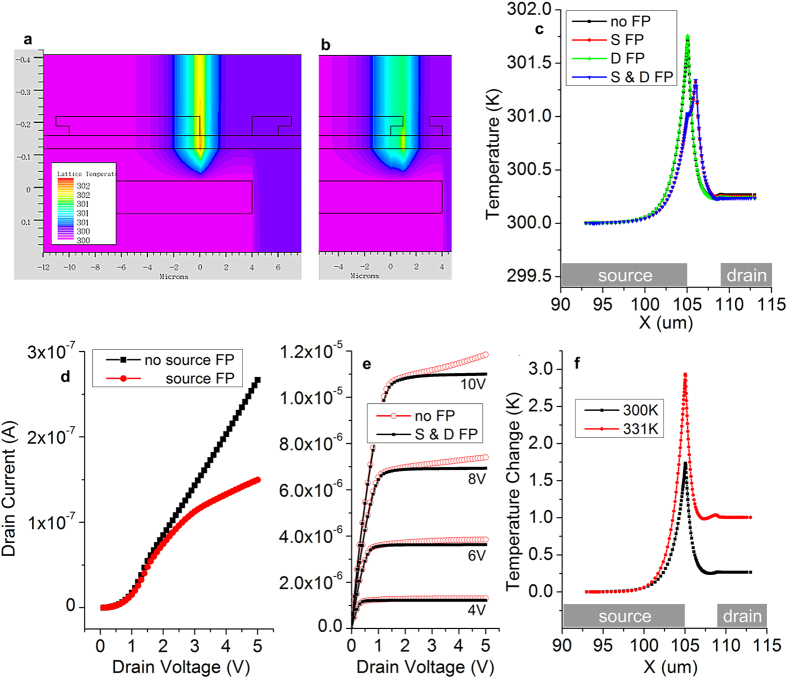
Effects of field relief structures built into electrodes and of ambient temperature on self-heating characteristics of polysilicon source-gated transistors (*V*_*G *_= 10 V, *V*_*D *_= 5 V, ϕ_B _= 0.35 eV). (**a**) device cross-section and temperature distribution when the gate is a constant-temperature heat-sink (300 K); (**b**) The same structure but with field-relief structures (field plates) built into the source and drain electrodes; (**c**) cut-line of temperature distribution showing the effect of source field plate: the hot-spot is translated away from the tip of the source and the maximum temperature is smaller. The effect of the drain field plate is negligible; (**d**) excess drain current against the reference (self-heating mechanism disabled in simulator) for devices with different source electrode architectures; (**e**) Output characteristics illustrating the effect of incorporating field plates into the source and drain electrodes; (**f**) The positive temperature coefficient of drain current results in higher current levels as ambient temperature increases. Consequently, self-heating increases, but peak temperature increase relative to ambient is low (no field plate).

Case II (gate as heat sink) leads to lower self-heating effects, on the order of 2 K, as presented in Fig. 3. Source and drain electrode shape was changed to compare rectangular contacts (Fig. 3a) with practical (design rule-obeying) metallizations which overlap the contact hole in the insulator, creating passive field relief^66^,^78^ structures (Fig. 3b). The drain-side field plate has no effect, but the source field plate extending into the source-drain gap moves the hot-spot away from the source contact edge. The hot-spot temperature is also lower than in the case without field plate. A cross-section of temperature taken at the semiconductor-insulator interface for the four possible combinations of field plates is shown in Fig. 3c. The higher temperature increase in the device without the field plate results in an larger increase of the drain current (positive TC) as illustrated by Fig. 3d. Figure 3e shows the shape of the corresponding output characteristics, as a visual comparison of the output conductance (saturated characteristic flatness).

Figure 3f illustrates the consequences of operating the device at higher ambient temperature: increased self-heating.

**Figure 4:**
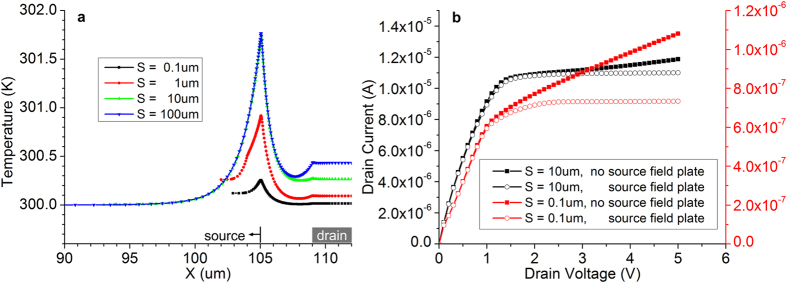
Effect of source electrode length (*S*) on the self-heating behaviour of source-gated transistors. (**a**) for short *S,* the current increases with *S*, thus the heating in the depletion region at the edge of the source also increases. For very long *S*, the current stops increasing with *S*, since the potential drop across the semiconductor far from the source edge closest to the drain becomes negligible, and heating (and temperature remain constant); (**b**) Output characteristics for *V*_*G *_= 10 V and two extreme values of *S*. Due to the different temperature dependence of the currents being injected from the edge and from the bulk of the source, the short-source device is more susceptible to changes to drain current due to self-heating. Incorporating a field plate lowers the local temperature at the tip of the source, restoring the low output conductance to the saturated current of the device with a short source.

The recently-described method^79^ of lowering the overall temperature coefficient of SGT drain current by increasing the source length, *S*, was applied to the simulated structure. Current from the “bulk” of the source has a lower temperature dependence than that injected at the edge of the source closest to the drain. In Fig. 4a, the net temperature at the semiconductor-insulator interface is plotted for four values of *S*, while in Fig. 4b, the effect of changing *S* on the output characteristics of devices with and without source plates is presented.

**Figure 5:**
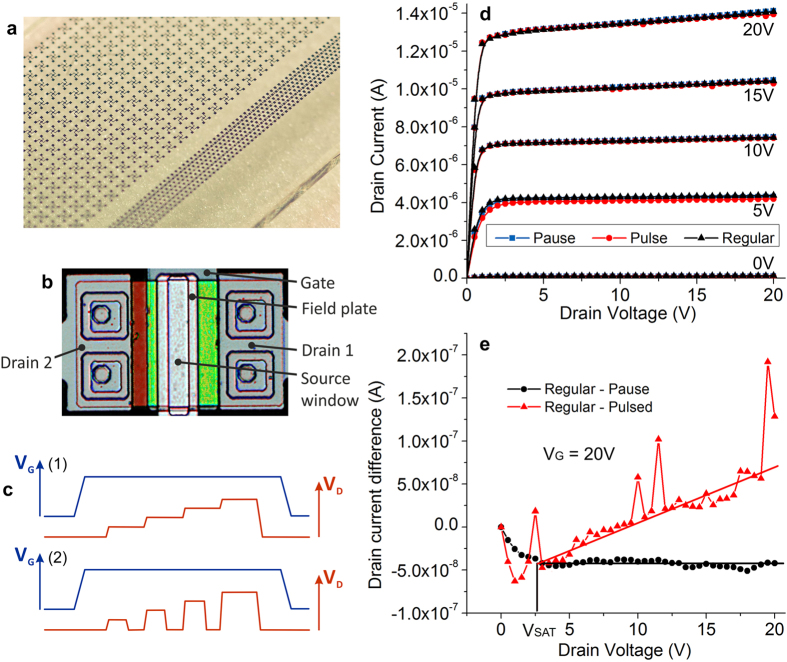
Measurements on fabricated polysilicon source-gated transistors confirm low self-heating effects. (**a**) Photograph of substrate (glass) with array of SGTs; (**b**) micrograph (top view) of typical fabricated SGT. Drain 1 is used during measurements, with Drain 2 floating; (**c**) Waveforms for measuring output characteristics: to measure an output curve, the gate voltage is set (step), then the drain voltage is changed (sweep) in equal increments for a “regular” measurement – (1), or pulsed, returning to zero between consecutive measurements (“pulsed” – 2), with a 500 ms pause is introduced between each pulse. To ensure that the effect measured is to do with heating rather than time-dependent processes (e.g. trapping), a version of (1) with a 500 ms pause between a *V*_*D*_ increase and current measurement was also performed (“pause”); (**d**) Output characteristics obtained by measuring with the three *V*_*D*_–changing methods; (**e**) In saturation, the “regular” and “pause” curves are identical, while the difference in current between the “regular” and “pulsed” measurements increases with *V*_*D*_, revealing the self-heating effect.

Polysilicon SGT output characteristics have been measured using three shapes of drain voltage (sweep) waveforms. The photograph of the glass substrate with an array of devices and the micrograph of a single device are shown in Fig. 5a,b, respectively. The main difference between the waveforms is illustrated in Fig. 5c. Figure 5d represents the measured output characteristics using the three input waveforms. Finally, Fig. 5e shows the additional current generated by self-heating as a function of drain voltage.

## Discussion

Polysilicon was chosen as the material system for this study as it allows the use of a low source barrier for high currents and maximum self-heating. Additionally, the kink effect due to impact ionization^80^,^81^,^82^,^83^,^84^, present in high mobility silicon TFTs and MIS^85^ structures, and albeit small in SGTs^66^,^67^ contributes to the total current and SH at high drain bias, making this material the most susceptible to self-heating effects.

However, due to the complexity of the calculation, the multi-physics algorithms did not converge when material defects, impact ionization and lattice heating were enabled simultaneously. For this reason, the defect statement was abandoned in favour of a reduction of carrier mobility throughout the semiconductor to model a crystalline material with polysilicon-like current capability. We deemed this to be a reasonable simplification, as the impact ionization model was kept enabled and likewise the essential lattice heating routine. The discrepancy with real polycrystalline material behaviour is largely related to transport in the accumulated channel. In SGTs, current is controlled in the source region, and the source-drain channel acts as a proportionally small series resistor, and its precise magnitude should not play a role in current control, just like source-drain gap, *d*, is not an essential SGT design parameter within a large range of values.

In the output characteristics we chose a relatively small maximum value for drain voltage, 5V. Previous studies, including measurements on polysilicon^66^,^67^, amorphous silicon^61^,^62^,^86^ and amorphous polymer^77^ devices have shown that the SGT architecture successfully suppresses kink effect for flat output characteristics up to at least |*V*_*D*_*| *= 10 V. For this study, we have focused on the low range *V*_*D*_, as one of the most likely applications for SGTs in an analog environment involves operation in saturation at *V*_*D*_ lower than *V*_*G*_ − *V*_*th*_, impossible for conventional FET structures.

The value for effective barrier height in the absence of bias, ϕ_B_^0^ was chosen as 0.35 eV: low enough to inject current at a level suitable for practical applications and simultaneously to contribute significantly to self-heating; but high enough to be the exclusive current-limiting and control mechanism (as opposed to the accumulated channel between the source and the drain in a standard FET).

The effects of heating are most pronounced in the structure with a thick insulating polymeric substrate which is a poor heat conductor and a single heat sink on the bottom of the substrate. Figure 2a shows the gradual divergence of curves obtained with lattice heating enabled and disabled as drain voltage is increased. For the range of *V*_*D*_ considered, it is important to note that at *V*_*G *_= 10 V the source is pinched off, but the drain is not (the accumulated channel is operating in the FET linear region). Changes in *V*_*D*_ above the source pinch-off voltage (*V*_*SAT1*_) are almost entirely dropped across the pinch-off region at the source, changing the shape of the depletion region to reach equilibrium, with small or no changes in drain current (see Supplementary Figure S1). The resistance seen by the current travelling horizontally through the pinched off area underneath the tip of the source, however, increases with the expansion of the source depletion region. Larger heat generation is observed at high *V*_*D*_ due to Joule heating (*P = R*^*2*^*I*), and the hot spot is in the region where resistance is highest: the source pinch-off region (Fig. 2b). Heating occurs, naturally, in the regions of the device with the largest current density, with the majority localized in the resistive source pinch-off region (Fig. 2c,d). The edge of the metal-semiconductor contact closest to the drain sees the most severe temperature increase. This is the most temperature-sensitive area of the device, as it corresponds to a metal-semiconductor contact in reverse bias and its reverse current has a large, positive temperature dependence. As a consequence, current injected by that area of the source increases with temperature, leading to the effect seen in Fig. 2a.

We have previously found that field relief plates at the source of SGTs have beneficial effects in suppressing drain field dependence of drain current^74^, including kink effect, up to comparatively high *V*_*D*_. These structures are normally part of contact design in photolithographically-defined top contacts. Incorporating a field plate into the source contact creates a pinch-off region at its tip and away from the edge of the source (Supplementary Figures S2 and S3). This resistive region screens the edge of the source from drain-induced electric field, but it also contributes to heat generation as drain current crosses it. The effect is a shift of the hot spot away from the highly temperature-sensitive edge of the source (Fig. 3a,b). A drain field plate, while easy to implement, has practically no effect on self-heating (Fig. 3c), largely due to the fact that at the drain voltages considered the drain is not pinched off. The drain current increase due to self-heating is significantly suppressed when a source field plate is included (Fig. 3e).

As the control mechanism in the area of the source closest to the drain (reverse-biased Schottky barrier) has a strong positive dependence on temperature, ambient temperature variations will also lead to changes in the magnitude of current injected at the source. Figure 3f shows the effect of raising the ambient temperature by 31 K. Total current passing through the pinch-off region at the source increases, leading to larger joule heat generation, higher temperature in the critical device region and ultimately even higher current injection from the edge of the source (positive feedback). The temperature increase due to self-heating is small (1 K for an ambient increase of 31 K) and, while somewhat detrimental to output characteristic shape, the effect is not enough to cause catastrophic thermal runaway.

A means of controlling the temperature sensitivity of SGT drain current is changing the length of the source, *S*. Depending on *S*, the dominant current injection mechanism changes^77^. The current from bulk of the source has a lower TC, and devices with long sources, in which the bulk injection dominates, should see smaller effects of self-heating on drain current. Figure 4a shows the increase in hot-spot temperature with *S*. This is to be expected, as larger S implies larger drain current. For very long *S*, however, the current saturates with *S*^77^,^87^. The lack of increase in current from *S *= 10 μm to *S *= 100 μm is reflected in the overlapping temperature cross-section plots. Despite the increase in hot-spot temperature, the long source device has little increase in drain current, as the majority of its current is injected from the low-TC bulk area of the source. The extremely short *S* (didactical) device, however, has significant modulation of drain current due to self-heating and the presence of a field plate greatly alleviates this problem by shifting the hot spot as discussed previously. Practically, moderate values of *S* in the few-microns to 10 μm region should produce good self-heating performance while being easily implemented with current technology and keeping gate overlap capacitance in a reasonable range.

Polysilicon SGTs (Fig. 5) have been fabricated on glass substrates according to the recipe in^67^,^88^. Measurement routines have been modified in an attempt to isolate the effect of self-heating on drain current. These devices have a built-in field plate and show excellent output characteristics up to *V*_*D *_= 30 V.

Regular measurements use a continuous drain sweep. The pulsed routine keeps the gate voltage constant for the duration of an output scan and asserts the drain voltage only for the duration of the measurement, with a 500 ms pause between data points (Fig. 5c). By keeping *V*_*G*_ constant, the trapping effects and large time constants can be eliminated as causes of differences seen between the “regular” and “pulsed” measurements, isolating the self-heating effects. The 500 ms pause was judged to be sufficient for heat dissipation between data point measurements. For completeness, a conventional drain sweep was performed, but with 500 ms pause between measurements and with *V*_*D*_ still asserted (“pause”).

Output characteristics (Fig. 5d) show the extent of the differences obtained while measuring with the three waveforms. Differences are minimal, even at high drain voltage. Figure 5e presents the findings. The “regular” and “pause” measurements show virtually identical values in saturation and no drain voltage dependence, denoting negligible time-dependent effects in the settling of the current between the regular measurement (tens of milliseconds) and the one including the 500 ms pause.

The difference between the “regular” and “paused”-type curves shows minimal drain voltage dependence, with a maximum current difference of approximately 0.75% at *V*_*G *_= 20 V and *V*_*D *_= 20 V. The current is larger in the “regular” curve. We deduce that the difference is due to self-heating and we relate the effect to the processes described above. Its magnitude is diminished due to lower current density in the fabricated device (source barrier approximately 50 meV higher); to a comparatively long source (10 μm); to the presence of an effective field plate; extending 2 μm over the edge of the source contact; and to ample metallic connections to all electrodes for improved heat transport. Self-heating plays a minimal role in polysilicon SGTs and is likely to be completely negligible in other, lower-current technologies. The only performance penalties are the slight reduction of intrinsic gain (proportional to (d*I*_*D*_/d*V*_*D*_)^−1^) and minuscule increased power dissipation due to the increase in drain current.

In conclusion, we have studied the effects of self-heating on the performance of polysilicon source-gated thin-film transistors. The temperature coefficient of drain current (TC) in SGTs with Schottky source contacts is positive and potentially very high, and could lead to thermal runaway and even device failure. Using Silvaco Atlas we performed multi-physics numerical simulations on a polysilicon structure. Two thermal scenarios were studied: in the first, the gate acted as the constant-temperature heat sink; in the second, theoretically worst-case scenario, none of the electrodes were heat-sinks. In neither case was self-heating more than a few Kelvin, localized in a hot-spot near the edge of the source closest to the drain.

Our findings suggest that self-heating does not significantly increase device temperature for the SGT, and thus the (temperature-dependent) drain current is largely unchanged during operation. Device optimizations can, however, reduce the effect of self-heating further. A field plate, incorporated in the source contact and standard practice for adherence to layout design rules in photolithographically defined top contacts to large-area silicon devices, can relieve the tip of the source from drain electric field but also shift the temperature hot-spot away from the source edge, lowering the temperature seen by this highly temperature-sensitive current injection area. Source length, *S*, can also be used as a parameter for lowering the effective temperature coefficient of drain current, as the bulk of the source is both less sensitive to temperature and experiences a lower temperature increase through self-heating.

We found that practically all self-heating occurs through Joule heating, suggesting that higher current density produces proportionally higher heat dissipation. For this reason, polysilicon was chosen due to its high mobility; for a low source barrier, the current density in the device is high, which in turns leads to increased self-heating. Lower mobility materials and high-barrier, low-current devices will experience still less self-heating.

Measurements on polysilicon devices confirm our simulation results. The measured self-heating effect on drain current is minimal, due to improved heat dissipation and somewhat lower current levels in the measured device.

Thermal runaway and device failure is unlikely. From a circuit design perspective, self-heating may cause modest current increase in devices operating at high currents in close proximity for prolonged periods. Local temperature differences through self-heating across a large-area circuit may lead to mismatched currents and output conductance. Judicious layout matching and proper device design (field plate, *S*) can all but eliminate these variations for most practical purposes.

Source-gated transistors are well suited to specialized use in high gain, low-power circuit blocks for a variety of analog and digital functions. With proper design, they can be very tolerant to process and operating condition variations, leading to reliable, consistent performance.

## Methods

### Device modelling

The Silvaco suite (Atlas v. 5.18.3.R; Athena v. 5.20.0.R) was used for source-gated transistor (SGT) device modelling and simulation.

The two-dimensional structure cross-section was defined using Athena (Fig. 1a). A bottom aluminium gate 100 nm thick, a 100 nm layer of silicon oxide which served as a gate dielectric, and a 40 nm silicon layer were successively defined on a 10 um-thick polymer substrate. The drain contact was self-aligned to the gate. The source completely overlapped the gate, as is convenient for SGT operation. The drain region was implanted with phosphorus (Athena parameters: dose = 1.0e15 energy = 4) to create the ohmic drain contact. Source (varying length, *S*) and drain (1 μm) contacts were defined on the semiconductor with and without overhangs (1 μm long, separated from semiconductor by 40 nm oxide), typical of top contact definition, to serve as field relief structures. Source-drain gap was *d *= 1 μm, typical of current low-cost lithographic technology. The whole structure was passivated with 200 nm silicon oxide. Device width is assumed *W *= 1 μm.

### Device simulation

Electrical and thermal properties were set in Silvaco Atlas prior to multi-physics simulation. Meshing in the *x* direction was set to 25 nm spacing at the source edge closest to the drain to increase the resolution in the region of interest where both self-heating and temperature-dependent current occur. For reduced simulation time, mesh spacing was relaxed in non-essential areas of the device, such as the edges of the design space and in the substrate.

The drain electrode was set to Aluminium and the contact was made ohmic through implantation in Athena. A source contact barrier was deliberately introduced through defining the source contact as Schottky with a nominal barrier (metal work function to semiconductor electron affinity) of ϕ_B_^0 ^= 0.35 eV. Gate work function was set to 5.2 eV to obtain a turn-on voltage around *V*_*G *_= 0 V.

Thermal properties were set to the defaults for the respective materials. Material conductivity, important for resistive heating estimation, is set through the mobility definition statements. Lattice heating was enabled as a means of simulating self-heating. Both Joule (resistive) heating and generation-recombination heating were extracted as two-dimensional overlays in the device cross-section at a number of operating points. Note that heating results are only valid in the semiconductor and all data in the insulating layers is spurious, resulting from meshing and calculation limitations^89^.

The worst case scenario was simulated by allowing only the bottom of the structure to act as heat sink. The device is separated from this constant (ambient) temperature heat sink by the thermally insulating plastic substrate (thermal conductivity: 0.15 Wcm^−1^K^−1^, thickness 25 μm). A more realistic but still challenging scenario was studied in which the gate acted as the constant temperature heat sink.

Initially, the semiconductor was set to default Atlas polysilicon parameters. However, upon enabling impact ionization and self-heating, convergence was difficult. The material was then modified to crystalline silicon with reduced mobility of 200 for electrons and 20 for holes. This is still a good approximation of the real self-heating effect, as the current is limited by the barrier.

Output and transfer characteristics were saved, together with the device snapshots at various biasing conditions (linear regime, saturation) and for a number of geometrical and electrical variations. A batch run consisted of different permutations of source length *S*, source work function ϕ_B_^0^, ambient temperature, heat sink position, and presence or absence of drain and source field plates.

### Fabrication

Polysilicon structures (Fig. 5a,b) fabricated on glass substrate according to^67^,^88^ were studied. Device width was W = 50 μm, source-drain gap *d *= 10 μm, source length *S *= 8 μm , semiconductor thickness *t*_*si *_= 40 nm, insulator thickness 200 nm oxide and 200 nm nitride. Cr and AlTi source/drain contacts were evaporated bulk doping, creating a 2 μm field plate separated by 120 nm oxide from the semiconductor. Bulk doping in the semiconductor was 0.5·10^12^ BF_2_.

### Measurement

The in-house measurement setup consisted of LabView-controlled Keithley 2400 source-measure units for setting drain and gate voltages and a Keithley 6458 picoammeter for measuring source current. The devices were probed with 5 um W needles on a Wentworth probe station with a heated, electrically isolated, motorized X–Y stage. Ambient temperature was 25C. The devices were exposed to the ambient fluorescent indoor light during measurement.

The control software was modified in order to make the effects of self-heating apparent. For tracing output characteristics, the conventional setup asserted a gate voltage which was kept constant during a drain voltage sweep from zero to maximum voltage. We call this the “regular” measurement sequence (Fig. 5c). The modified “pulsed” sequence involves asserting the drain voltage for the duration of each data point measurement, then returning it to zero for 500 ms to allow induced heat to dissipate. Finally, a dummy mode denoted “pause” was used in which the drain voltage is stepped as per the “regular” scheme without returning to zero after each measurement, but a 500 ms pause was introduced after each data point with drain voltage applied.

## Additional Information

**How to cite this article**: Sporea, R. A. *et al.* Self-Heating Effects In Polysilicon Source Gated Transistors. *Sci. Rep.* **5**, 14058; doi: 10.1038/srep14058 (2015).

## Supplementary Material

Supplementary Information
