# Supplementary material for: Self-Heating Effects In Polysilicon Source Gated Transistors
Source: Sci Rep. 2015 Sep 9;5:14058. doi: 10.1038/srep14058 (PMC4650605; doi:10.1038/srep14058)
Supplement: Supplementary Information [file srep14058-s1.pdf]

Self-Heating Effects In Polysilicon Source Gated Transistors

R. A. Sporea, T. Burridge and S. R. P. Silva

Supplementary material

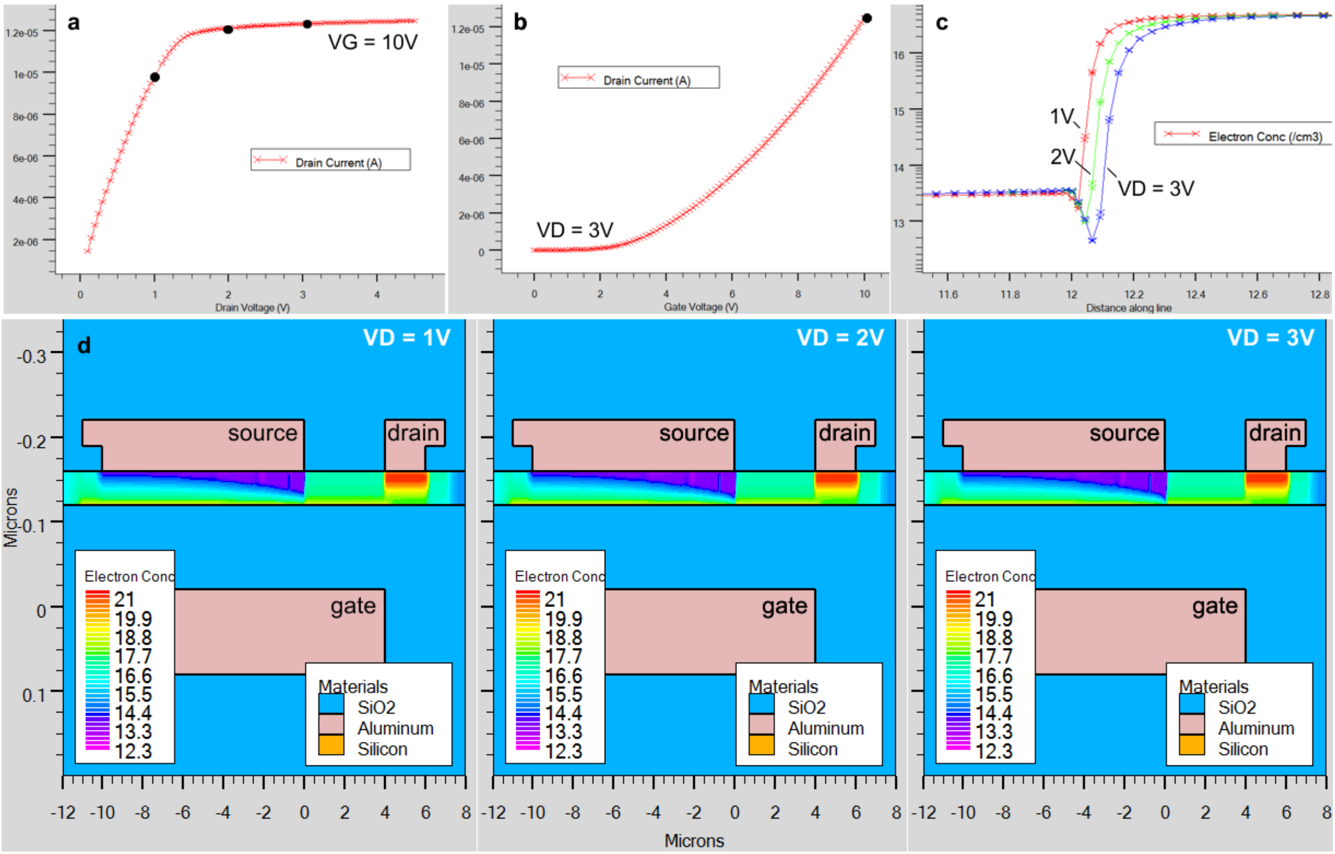

Figure S1. Electron concentration changes in the source area with drain voltage variations. a) output characteristics illustrating the values of drain voltage considered: 1V (before saturation), 2V and 3V (source pinched off but semiconducting channel in linear region); b) transfer characteristics, Vth ≈ 3V; c) electron concentration at the edge of the source electrode (x = 12μm) for three values of V_D. The depletion region changes shape to accommodate the change in biasing condition; d) corss-sections of electron concentration in the semiconductor for the three values of V_D.

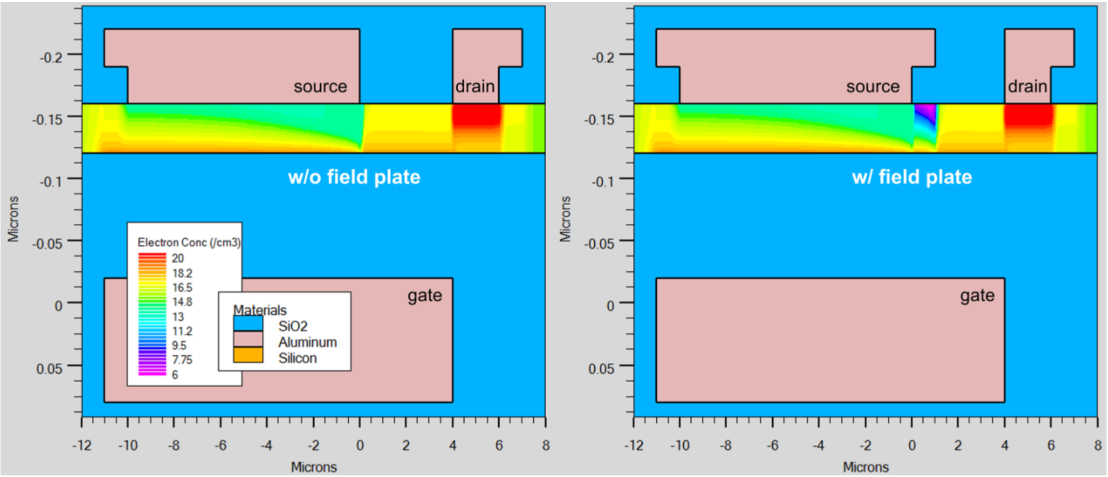

Figure S2. Device cross sections showing the distribution of electron concentration in the semiconductor layer for at V_G=10V, V_D = 5V, for a device without (left) and with (right) source field plate.

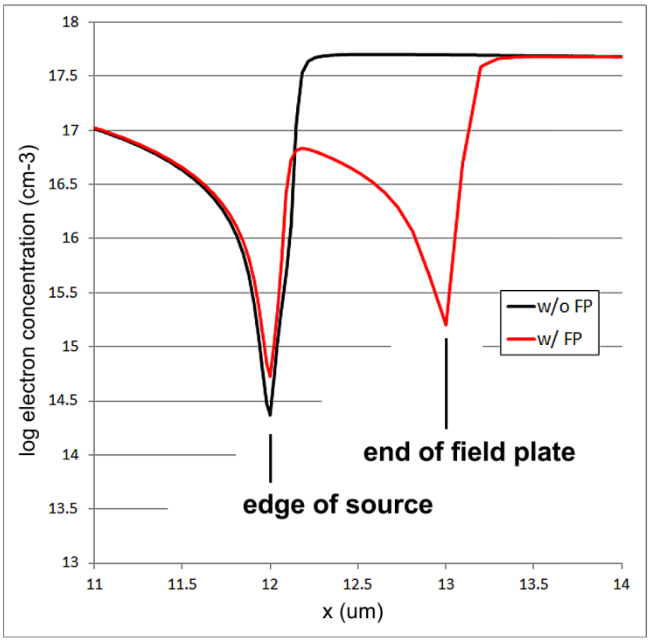

Figure S3. Electron concentration distribution at the insulator interface for V_G=10V, V_D = 5V. The presence of a source field plate reduces the depletion at the edge of the source, increasing its conductivity and decreasing heat generation at that spot.
